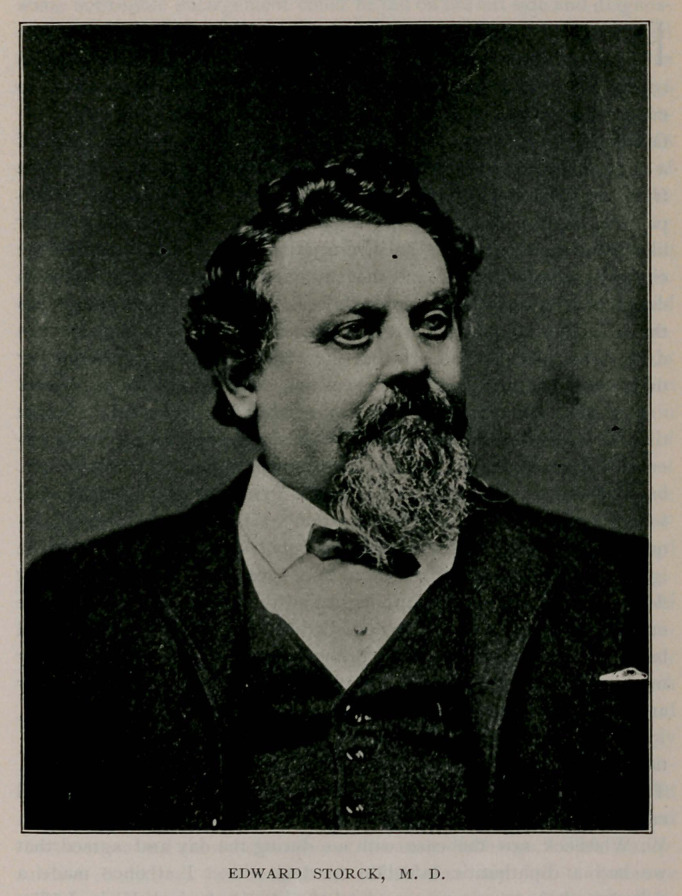# A Century of Medical History in the County of Erie.—1800–1900

**Published:** 1898-06

**Authors:** William Warren Potter

**Affiliations:** Buffalo, N. Y.


### A CENTURY OF MEDICAL HISTORY IN THE COUNTY OF ERIE.—1800–1900.

By WILLIAM WARREN POTTER, M. D., Buffalo, N. Y.

*Pioneer Physicians—Medical Societies—Medical Colleges—Hospitals—Medical Journals—Medical Officers of the Civil War—Women Physicians—History of Homeopathy—Individual Members of the Profession.*

[*Continued from the May edition.*]

1844—James Allen, Gilbert McBeth, William Treat, James B. Samo, Isaac Parsell, Samuel S. Prudden, Samuel G. Bailey, John Hauenstein, John S. Trowbridge, George N. Burwell and Jesse F. Lock.

The contribution of this year, too, contains a group of physicians, many of whom attained prominence. William Treat, who came to Buffalo from Maine, was elected to membership in 1844, and became president in 1860. He was a man of literary attainments and contributed several valuable papers to the Buffalo Medical Journal. In July, 1861, after the battle of Bull Run, he went to Washington and repaired to Fort Runyon, an earthwork on the Virginia side of the Potomac near the end of Long Bridge, where he was assigned to duty in caring for the wounded as they came from the battlefield. Afterward he also assisted at the city hospital in rendering a similar service. At a meeting of the Buffalo medical association, held in August, 1861, he gave an interesting account of his observations and, singularly and sadly, died before the month ended. Dr. Treat commanded the respect of his colleagues as well as a large clientèle.

Two members were chosen in 1844, one of whom is still living, who were active participants in the work of the society for more than forty years. James B. Samo, a native of New Jersey, one of these, was elected librarian in 1852 and became president in 1862. He was local marine hospital surgeon from 1853 to 1859 and he enjoyed the respect and confidence of his professional friends during his long

VIEW OF BUFFALO, 1845.

period of membership in the society. He was librarian for forty years—namely, from 1852 to 1892. He died March 12, 1897, aged 85 years.

John Hauenstein, still living though retired from active practice, became a member in 1844, and was chosen president in 1881. He has read many excellent papers before the society, one of the latest on the First uses of anesthetics in Buffalo, at the seventy-fifth anniversary meeting of the society, January 14, 1896. This paper was published in the March, 1896, edition of the BUFFALO MEDICAL JOURNAL. Another contribution, entitled A résumé of fifty years' obstetric practice, was published in the same journal in its issue for June, 1897. Dr. Hauenstein, having ceased the active practice of a profession that he has so long adorned, lives in the enjoyment of good health and the respect and confidence of a large community.

Samuel G. Bailey also united with the society in 1844. He had been a pupil of Dr. James P. White and was elected treasurer in 1852, holding to and including 1855. He ceased to be a member in 1856.

John S. Trowbridge, son of Josiah Trowbridge, was one of the accessions in 1844 and in 1845 he was chosen a censor of the society, continuing as such during 1846. In 1848 he was elected secretary, holding office until 1851. At the annual meeting of the society, January 12, 1869, Dr. Trowbridge read a biographical sketch of his father, Josiah Trowbridge, which was also read a week later before the Buffalo Historical Society and sent out with the February, 1869, issue of the BUFFALO MEDICAL JOURNAL. About the year 1874 Dr. Trowbridge retired from the practice of his profession and established a drug store at the corner of Niagara and Carolina streets. He died April 2, 1886, aged 69 years.

George N. Burwell was one of the accessions in 1844 who acquired fame in the profession and who for many years was active in the councils of the society. He had an extensive following among rich and poor and may justly be rated as one of Buffalo's most successful physicians. He died May 15, 1891, aged 71 years.

Officers for 1844—President, William K. Scott; vice-president, Orlando Wakelee; secretary, James P. White; treasurer, H. N. Loomis; librarian, Josiah Trowbridge; censors, F. L. Harris, H. H. Bissell, C. H. Raymond, Isaac Parsell, George H. Lapham; delegate to state society, Alden S. Sprague.

1845—Frank Hastings Hamilton, —— Rogers,[1] Caleb H. Austin.

Frank Hastings Hamilton, who joined the society in 1845, came to Buffalo from Geneva, where he had been teaching anatomy and surgery for several years. In 1846 he was elected professor of surgery in Buffalo Medical College, which chair he held until 1860. He

1. Christian name does not appear on the records.

was a censor of the society from 1851 to 1856 inclusive; was elected vice-president in 1856 and president in 1857. In 1851 he became a permanent member of the state society and was elected president of the same in 1856. During the fifteen years of his residence at Buffalo Dr. Hamilton was a constant contributor to the BUFFALO MEDICAL JOURNAL, in which he published his early fracture tables and papers relating to deformities after fractures, contributions that laid the foundation for his future classic treatise on fractures and dislocations, a work that has been translated in several foreign languages. In 1860 Dr. Hamilton removed to New York, becoming professor of surgery at the Long Island College Hospital, was chosen to the same chair at Bellevue Hospital Medical College upon its organisation in 1861. Dr. Hamilton died August 11, 1886, at the age of 73. Perhaps no man of his time contributed more to maintain the *esprit de corps* of the profession of medicine than did this educated, accomplished and upright surgeon.

FRANK HASTINGS HAMILTON, M. D.

It was during 1845 that the BUFFALO MEDICAL JOURNAL was established, and we find in the records of the society a subscription order for six copies, by which act the society testified its loyal support of the JOURNAL.

Officers for 1845—President, Orlando Wakelee; vice-president; F. L. Harris; secretary, Charles Winne; treasurer, Horatio N. Loomis; librarian, Josiah Trowbridge; Censors, Austin Flint, George N. Burwell, S. F. Mixer, John S. Trowbridge and T. T. Lockwood.

1846—G. D. Stevens, Archibald S. Clark, Daniel Devening, Sidney W. Cole.

At the annual meeting, January 13, 1846, Dr. Josiah Trowbridge offered a resolution instructing the committee on books to invest the money in the hands of the treasurer after June 15th in the purchase of rare and valuable books, this action being the foundation of a library.

Officers for 1846—President, Francis L. Harris; vice-president, Isaac Parsell; secretary, Charles Winne; treasurer, Horatio N. Loomis; librarian, Josiah Trowbridge; censors, J. B. Pride, John S. Trowbridge, George N. Burwell, William K. Scott, S. F. Mixer; delegate to the state society, Alden S. Sprague.

1847—Joseph Peabody, Walter Cary, James M. Newman, Ewald Beckendorf, Phineas H. Strong and S. W. Sole.

James M. Newman, who joined the society in 1847, had been a student of Dr. James P. White. He held the office of secretary from 1852 to 1859. The records of the society during that period are among the best in the volume. Dr. Newman was appointed health physician of Buffalo in 1854 and he became attending physician at the Buffalo General Hospital in 1858. He removed from Buffalo in 1859 and died in 1860, lamented by everyone who knew him. He was a young man of rare promise and left a name to be revered and an example to be emulated.

Phineas H. Strong, a native of Vermont, came to Buffalo in 1846, was elected to membership in 1847, to the presidency in 1853, and was chosen a delegate to the state society in 1855. He became a permanent member of the latter in 1859. He was appointed health physician of Buffalo in 1859, and following his appointment submitted the question of accepting it at a less compensation than that fixed by the fee bill to a vote of the society. Dr. Strong was an occasional contributor to the BUFFALO MEDICAL JOURNAL. He was appointed professor of medicine at Howard University, Washington, D. C., soon after its organisation, which chair he held for three years. He died at Buffalo, February 10, 1890, aged 72 years.

Walter Cary, a son of Trumbull Cary, was born at Batavia, December 21, 1818. He received his academic degree at Union College in 1839 and took his doctorate degree from the University of Pennsylvania in 1843. After serving a term in Blockley Hospital he went abroad for study. On his return he established himself in practice and so continued for about ten years, a large part of the time as a partner of Dr. Charles Winne. He then retired, living in ease and in the cultivation of his friendships and tastes. He died at Marseilles, France, November 1, 1880, aged 62 years. His body was cremated by his direction and his ashes were interred at Forest Lawn, Buffalo.

Officers for 1847—President, Isaac Parsell; vice-president, Charles H. Austin; secretary, George N. Burwell; treasurer, Josiah Barnes; librarian, Josiah Trowbridge; censors, James B. Samo, S. G. Bailey, Charles H. Wilcox, S. F. Mixer and J. B. Pride.

1848—J. P. Dudley, James E. King, Charles House, Carlo Schmidt, Joseph Felegmacher.

At the annual meeting, held January 11, 1848, Dr. William Treat, from a committee previously appointed to collect the names of regular and irregular practitioners of medicine, made a report. He presented the names and locations of seventy regular, thirty-two irregular and twelve undetermined practitioners in the county. In the city of Buffalo alone there were thirty-eight regular, twenty-one irregular physicians and four whose mode of practice was not determined.

Dr. Walter Cary, who had been appointed orator of the day, was not present. Dr. Cary was, however, appointed a delegate to the American medical association. His associates were Drs. Bryant Burwell and Alden S. Sprague.

Officers for 1848—President, C. H. Austin; vice-president, Charles Winne; secretary, John S. Trowbridge; treasurer, Josiah Barnes; librarian, Josiah Trowbridge; censors, Bryant Burwell, Horatio N. Loomis, Erastus Wallis, William Treat, H. H. Bissell; delegate to the state society, Horatio N. Loomis.

1849—Charles W. Harvey, Cornelius C. Wyckoff, Edward Mackey, Henry D. Garvin, William King, J. J. C. Haxsteen, L. P. Dayton and John D. Hill.

Cornelius Cox Wyckoff, who joined the society in 1849, is a native of Romulus, N. Y., and located at Buffalo in 1848. He was president in 1858; permanent member of the state society in 1867 and a member of the state board of censors from 1870 to 1883. He has been attending physician to the Buffalo general hospital since 1858. Dr. Wyckoff is still engaged in active practice and has attained high standing in the profession, while at the same time he enjoys the confidence of all who know him, his circle of acquaintance being very large. In 1898 he was appointed by Mayor Diehl a park commissioner.

Charles W. Harvey, who joined the society in 1849, was for many years a successful dentist in Buffalo, though he always kept in touch, at least during the years of his active life, with the guild of medicine. The name of his son, Dr. Leon F. Harvey, is still borne on the list of active members, though he lately removed to Denver, Colorado.

L. P. Dayton, who joined during 1849, was vice-president in 1858 and president in 1859. He was mayor of Buffalo in 1874-75 and is still engaged in the practice of his profession, holding the esteem of his colleagues and of the many people who know him.

John D. Hill, who joined the society in 1849, was expelled from membership at the annual meeting June 9, 1855. Subsequently he was restored to membership on an order of the court and was elected president of the society in 1888. Dr. Hill acquired a large practice and was respected by the community in which he lived for so many years. He died February 27, 1892, in the seventieth year of his age, lamented by a large circle of friends.

Officers for 1849—President, Erastus Wallis; vice-president, Charles H. Wilcox; secretary, John S. Trowbridge; treasurer, Josiah Barnes; librarian, Josiah Trowbridge; censors, Alden S. Sprague, George N. Burwell, James M. Newman, Horatio N. Loomis, William Treat; primary board, Horace M. Congar, Walter Cary and H. W. Barrett.

The duty of the primary board was to examine and certify to the preliminary acquirements of pupils about to begin the study of medicine. Here this society took the initiative in an important movement that resulted years afterward in establishing the principle by statutory law.

1850—E. P. Gray, L. J. Ham, Patrick Flood, J. E. Camp, Hugh B. Vandeventer, James S. Hawley, S. E. S. H. Nott, George Johnson, O. H. Needham.

L. J. Ham, who joined the society in 1850, came to Erie county from Maine in 1846, locating at Williamsville. He was elected president in 1852, but removed to South Bend, Ind., in 1859. He served during the war as surgeon of the 48th Indiana volunteers and was chairman of the operating board of surgeons of the 7th division of the 17th army corps in 1863-64. He also served as medical director of the 17th army corps under General McPherson. In 1871 he sent his portrait to the society with an autobiographical sketch, and on motion of Dr. Storck the society presented its thanks to Dr. Ham, wishing him many years of happiness and success.

S. E. S. H. Nott was a prominent physician at Hamburg for many years and he was elected one of the coroners of Erie County.

E. P. Gray was in active practice in Buffalo for several years, but removed west and died at St. Joseph, Mo., August 9, 1872.

Hugh B. Vandeventer was appointed demonstrator of anatomy at Buffalo Medical College in 1860. He subsequently removed to Long Island, where he died in 1890.

Officers for 1850—President, Charles H. Wilcox; vice-president, George N. Burwell; secretary, John S. Trowbridge; treasurer, Josiah Barnes; librarian, Josiah Trowbridge; primary board, Walter Cary, James M. Newman and H. W. Barrett; censors, Alden S. Sprague, J. E. Camp, J. B. Samo, H. N. Loomis, William Treat.

1851—Charles C. Jewett, Sandford Eastman, P. Barber and William Gould.

Sandford Eastman, who joined the society in 1851, was elected president in 1861. He was professor of anatomy in Buffalo Medical

College from 1859 until 1870, during which time he was also surgeon at the Buffalo general hospital and the hospital of the Sisters of Charity. He was appointed health physician of Buffalo, serving for several years. He acquired a large practice, was respected by all who knew him, and died January 8, 1874, aged fifty-three years.

Officers for 1851—President, Alden S. Sprague; vice-president, Horatio N. Loomis; secretary, Gorham F. Pratt; treasurer, Josiah Barnes; librarian, Josiah Trowbridge; primary board, George N. Burwell, E. P. Gray and J. E. Camp; censors, Frank H. Hamilton, Bryant Burwell, John D. Hill, John Hauenstein and J. D. Garvin.

1852—John C. Dalton, Jr., M. B. Norton, Hugh McVean, A. S. Griswold, Charles H. Barber, John Root, Ernest G. Pussikofer and Orlando K. Parker.

John Root was a prominent physician in Buffalo for many years, during a portion of which time he held the office of health physician. He removed to Batavia in 1858, where he acquired a large practice and died November 29, 1876, aged fifty-two years.

Orlando K. Parker, who joined the society in 1852, was elected president in 1869, and acquired fame as a practitioner of medicine in the town of Clarence. He died November 16, 1872, aged forty-six years.

John C. Dalton, Jr., the famous physiologist, never held office in the society, but his name deserves special mention in connection with his celebrity as a teacher of his chosen specialty. He taught physiology in Buffalo medical college for several years, then removed to New York, where he died February 12, 1889, aged sixty-four years.

Officers for 1852—President, L. J. Ham; vice-president, P. H. Strong; secretary, James M. Newman; treasurer, S. G. Bailey; librarian, James B. Samo; primary board, Sandford Eastman, J. E. Hawley and William Ring; censors, Frank H. Hamilton, John G. House, William Van Pelt, H. M. Congar and William Treat.

1853—E. D. Merriam, Alfred S. Spearman, J. J. Edmonds, Edward E. W. Gail, John Boardman, Ellery P. Smith, Benajah T. Whitney, John A. Jeyte, Joseph R. Smith.

John Boardman, who entered the society in 1853, had been a student of Prof. Frank H. Hamilton, and was elected president in 1868. He was sent as a delegate to the state society in 1855, and became permanent member thereof in 1862. In 1864 he represented the medical society of the state of New York in the National Quarantine and Sanitary Convention. Dr. Boardman has been a frequent contributor to the BUFFALO MEDICAL JOURNAL and assisted Dr. Hamilton in preparing his fracture tables, besides doing original work in that and other branches of surgery. In 1854 he was elected demonstrator of anatomy in Buffalo medical college and became attending surgeon at the hospital of the Sisters of Charity. Dr. Boardman still resides in Buffalo, where he has enjoyed for many years a very large practice among the most substantial citizens, though he has now retired from his active labors.

E. D. Merriam joined the society in 1853. He now resides at Conneaut, O., still pursuing the active practice of his profession, enjoying the confidence of a large clientèle.

Joseph R. Smith, who became a member in 1853, entered the regular army as assistant surgeon, and during a portion of the civil war served as assistant on the staff of the surgeon-general, U. S. Army, at Washington. He is now on the retired list of the army with the rank of colonel and resides at Philadelphia.

SANFORD B. HUNT, M. D.

The society gave its first annual dinner, June 14, 1853, at the Clarendon hotel, at 3 o'clock p. m. This was an interesting event that had been looked forward to for some time in pleasant anticipation. After a few years the custom was discontinued much to the regret of many who remember the occasions as delightful reunions.

Officers for 1853—President, Phineas H. Strong; vice-president, John G. House; secretary, James M. Newman; treasurer, S. G. Bailey; librarian, Josiah Trowbridge; primary board, Sandford Eastman, William Ring, J. E. Hawley; censors, Frank H. Hamilton, James B. Samo, William Van Pelt, William Treat and H. M. Congar.

1854—Sanford B. Hunt, Charles L. Dayton, T. W. Wood, Thomas F. Rochester, Richard W. Nelson, C. C. F. Gay, Austin W. Nichols, Frederick W. Gardner, C. B. Hutchins, Charles B. Richards, Edward Storck, William A. Newell and Joel Underhill.

Sanford B. Hunt, who became a member in 1854, during the same year was appointed professor of anatomy at the Buffalo Medical College. In 1853 he became associate editor of the BUFFALO MEDI-

CAL JOURNAL and in 1855 the magazine passed into his hands as sole owner and editor. At the semi-annual meeting, June 13, 1854, Dr. Hunt was the orator of the society and his subject was Cranial characteristics and powers of human races. This paper was published in the BUFFALO MEDICAL JOURNAL and attracted great attention. In February, 1855, Dr. Hunt delivered the valedictory address to the graduating class of the Buffalo medical college. This, too, was published in the BUFFALO MEDICAL JOURNAL and was a model in rhetoric, metaphor and diction. Dr. Hunt was a ready writer and did much to improve the literary taste of the medical profession. He was elected superintendent of public schools in 1858 and was also city editor of the *Buffalo Commercial* until 1861. During the latter year he entered the army as surgeon of United States volunteers, serving to the end of the war. He died at Irvington, near Newark, N. J., April 26, 1884, and his remains were brought to Buffalo for interment. A further notice of Dr. Hunt is given under the title of medical journals.

Thomas F. Rochester came to Buffalo from New York in 1853, and joined the county society in 1854. He was chosen professor of the principles and practice of medicine and of clinical medicine at Buffalo medical college on the resignation of Dr. Flint in 1853. Dr. Rochester became a permanent member of the state society in 1870, and was president in 1875. He occupied a prominent position in the professional as well as in the public affairs of Buffalo, taking specially active interest in the Buffalo Fine Arts Academy, of which he was president for many years. Dr. Rochester did a very large consulting practice throughout western New York, and maintained his activity up to within a few months of his death, which occurred May 27, 1887, when he was sixty-three years of age. A further reference to Dr. Rochester will be found under the head of medical colleges.

C. C. F. Gay, who joined the society in 1854, was a native of Massachusetts, and located at Byron, Genesee county, in 1847. He came to Buffalo in 1861, and served as president of the society in 1865. He was made permanent member of the state society in 1861, and was consulting surgeon at the Buffalo general hospital for many years. In 1878 he was chosen surgeon-in-chief of the Buffalo surgical infirmary, and in 1883 became professor of clinical and operative surgery at the medical department of Niagara university. The later years of his life were devoted to the practice of surgery, in which he acquired skill and fame. Dr. Gay died March 27, 1886, aged sixty-four years.

Edward Storck, who also joined the society in 1854, was appointed a member of the Union Defense Committee in 1861, and afterward served as surgeon at Fort Porter during the organisation of troops for the field. He was president in 1878, and served as chairman of the board of censors from 1880 to 1892—twelve years—when he resigned the office. During the entire period of his service quacks and irregulars had a sorry time in Buffalo, for Dr. Storck pursued them with all the energy that the law permitted. At the time of his resignation the society tendered him a vote of thanks for his faithful and meritorious services. He published a sketch of the work of the board during his administration in the BUFFALO MEDICAL JOURNAL, July, 1896. He was instrumental in securing legislation favorable to the society as well as in preventing that which would prove adverse to its interests. Dr. Storck acquired a large practice. His death occurred July 26, 1897, when he was 66 years of age.

THOMAS F. ROCHESTER, M. D.

Officers for 1854—President, John G. House; vice-president, James P. White; secretary, James M. Newman; treasurer, S. G. Bailey; librarian, J. B. Samo; primary board, Sandford Eastman, William Ring, James S. Hawley; censors, Frank H. Hamilton, J. B. Samo, William Treat, William Van Pelt and H. M. Congar; delegates to the state society, Thomas F. Rochester, H. M. Congar, James P. White and John G. House.

1855—J. C. Gay, Julius F. Miner, Edward Tobie, George Abbott, Samuel T. Hance and D. W. Hershey.

Julius F. Miner, who joined the society in 1855, was a decided accession to its membership. He reëstablished the BUFFALO MEDICAL JOURNAL, 1861, and in 1867 he was appointed professor of ophthalmology and surgical anatomy at the Buffalo medical college, a title that was changed in 1870 to professor of special and clincal surgery. He became a permanent member of the state society in 1869, and was president of the county society in 1870. Dr. Miner was a skilful surgeon, one of the most amiable of men, and a useful citizen. He was especially endeared to his pupils, who were numerous and who manifested their attachment to him on every and all occasions. He died November 6, 1886, aged 63 years.

CHARLES C. F. GAY, M. D.

At the annual meeting of the society, June 9, 1855, Dr. Edward Storck stated that the reputable German medical practitioners of the city had formed a society for their own benefit and for their protection against quackery as practised by unqualified practitioners among the German population; that the members of the said society were desirous of becoming legalised practitioners of medicine and of uniting with the Erie county medical society. He desired information as to the necessary steps to be taken to accomplish these objects. On motion of Dr. Charles H. Wilcox a committee was appointed to confer with the German society and to furnish information and assistance in accomplishing the objects sought. This action led to the adjustment of the relations between the two groups of physicians at interest.

EDWARD STORCK, M. D.

Officers for 1855—President, James P. White; vice-president, William Van Pelt; secretary, James M. Newman; treasurer, S. G. Bailey; librarian, James B. Samo; primary board, Sandford Eastman, William Ring, James S. Hawley; censors, Frank H. Hamilton, James B. Samo, William Treat, William Van Pelt and H. M. Congar.

(*Continued next month.*)

## Figures and Tables

**Figure f1:**
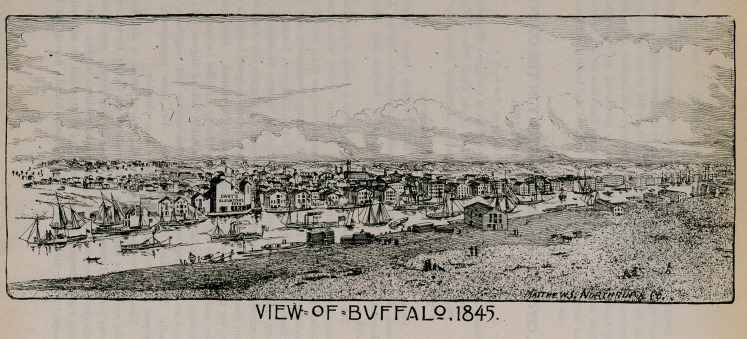


**Figure f2:**
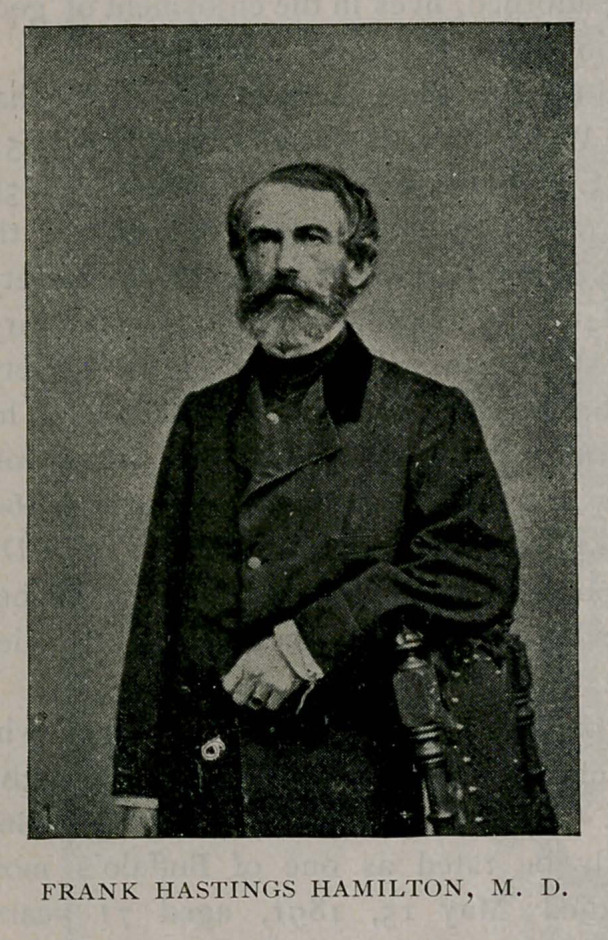


**Figure f3:**
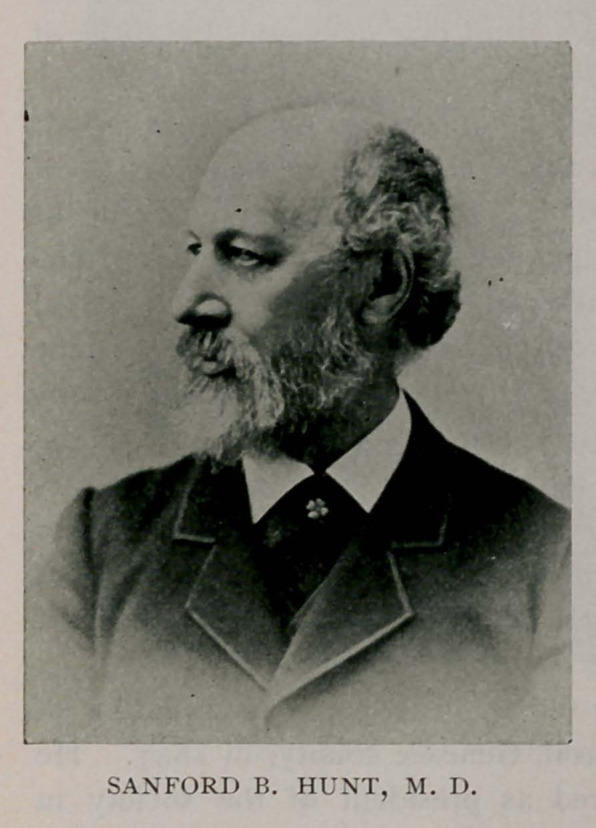


**Figure f4:**
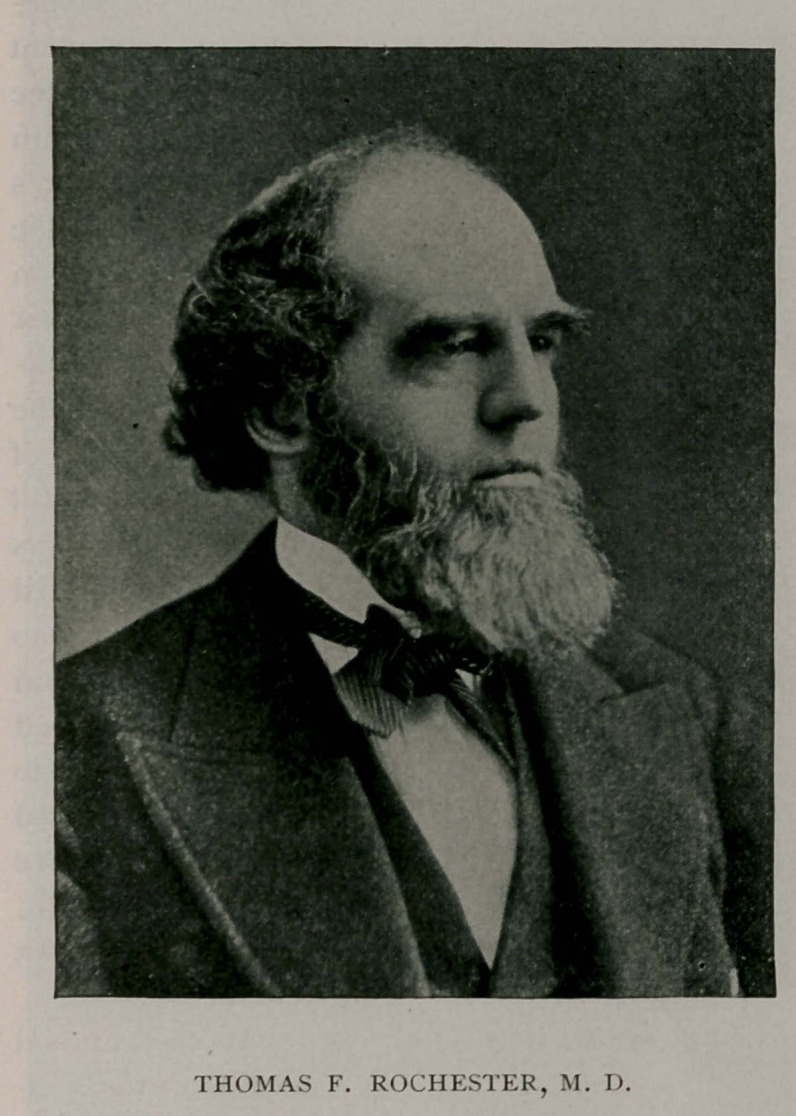


**Figure f5:**
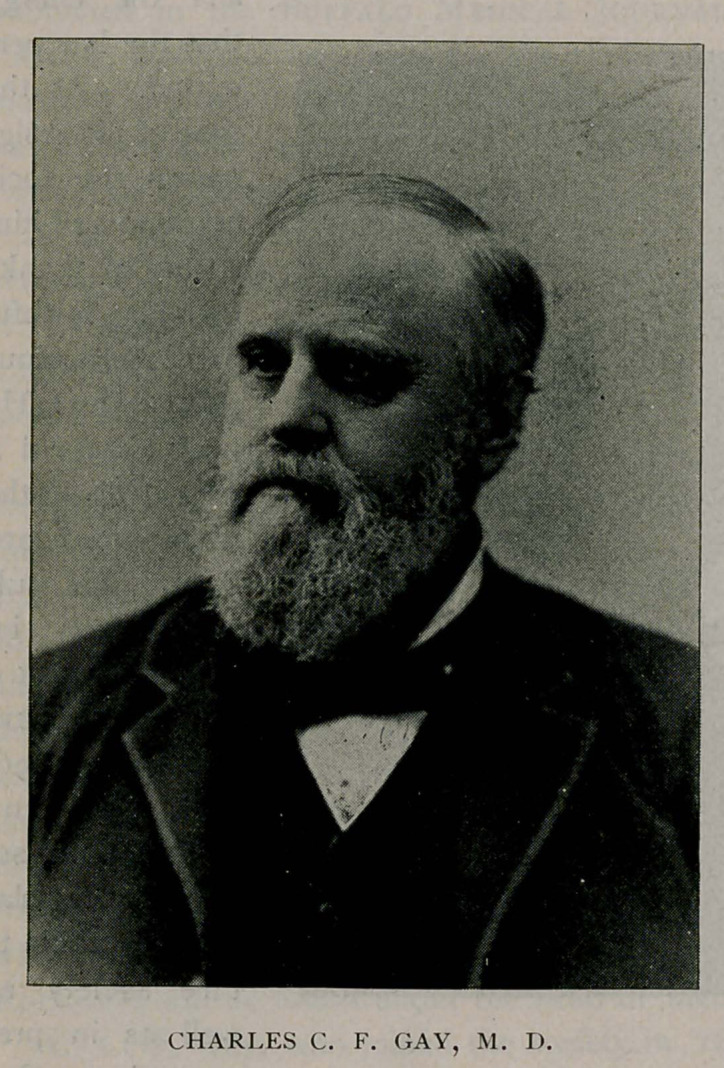


**Figure f6:**